# Nontyphoidal *Salmonella* as a Cause of Mediastinal Abscess after Aortic Valve Replacement: A Case Report and Review of Postoperative *Salmonella* Infections

**DOI:** 10.1155/2018/6758672

**Published:** 2018-11-26

**Authors:** Natasha Spottiswoode, Michael J. Peluso, Tobias Deuse, Jennifer M. Babik

**Affiliations:** ^1^College of Physicians and Surgeons, Columbia University, New York, NY, USA; ^2^Division of Infectious Diseases, Department of Medicine, University of California San Francisco, San Francisco, CA, USA; ^3^Division of Adult Cardiothoracic Surgery, Department of Surgery, University of California San Francisco, San Francisco, CA, USA

## Abstract

**Background:**

Nontyphoidal *Salmonella* (NTS) is a pathogen that causes several human clinical illnesses, most commonly gastroenteritis. Focal infections are rare and are generally reported in the gastrointestinal and genitourinary systems. Very few studies have reported NTS abscess as a postoperative complication.

**Case report:**

We describe an elderly patient who developed NTS bacteremia and mediastinal abscess after aortic valve replacement.

**Conclusions:**

This report describes an extremely rare occurrence of an NTS mediastinal abscess complicating a surgical procedure. The patient may have acquired the pathogen from a snake kept as a family pet and likely developed bacteremia followed by seeding of the surgically damaged tissues.

## 1. Case Report

An 86-year-old man with a medical history of coronary artery disease and severe aortic stenosis presented with fatigue, shortness of breath, and chest pain. The patient had undergone aortic valve replacement with a bioprosthetic Intuity valve 25 days prior to presentation. His postoperative course was complicated by excessive bleeding from chest tubes that necessitated a mediastinal washout and mild volume overload treated with diuretics. His chest tubes and pacing wires were removed on postoperative day (POD) 5 without issue, and he was discharged on POD 7.

The patient was noted to be doing well at follow-up appointments with his geriatrician on POD 13 and with the cardiothoracic surgery clinic on POD 20. However, on POD 24, the patient presented to the emergency department with several days of fatigue, subjective shortness of breath, and chest pain. He described his chest pain as worse at night and when lying flat. He complained of associated confusion and weakness, both of which were unusual for him. The patient's review of systems was otherwise negative, and he denied any other constitutional, respiratory, gastrointestinal, urinary, rheumatologic, or dermatologic symptoms.

**Figure 1:**
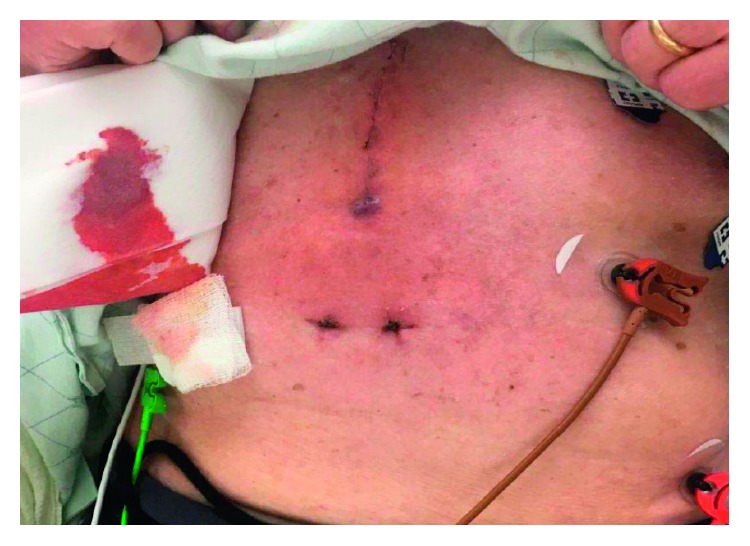
Skin exam on initial presentation, showing erythema at the inferior margin of the wound.

In the emergency department, he was febrile to 102 degrees Fahrenheit, and his exam was notable for a warm, erythematous, and tender epigastrium without obvious wound dehiscence (Figure 1). Notable labs included sodium 124 mmol/L, creatinine 1.5 mg/dL (baseline 1.2–1.3 mg/dL), AST 47 U/L, ALT 31 U/L, alkaline phosphatase 87 U/L, total bilirubin 1.1 mg/dL, and white blood cell count 6.4 × 10^9^ cells/L. Because of concern for wound infection, as well as his hypovolemic hyponatremia, he was admitted to the hospital.

**Figure 2:**
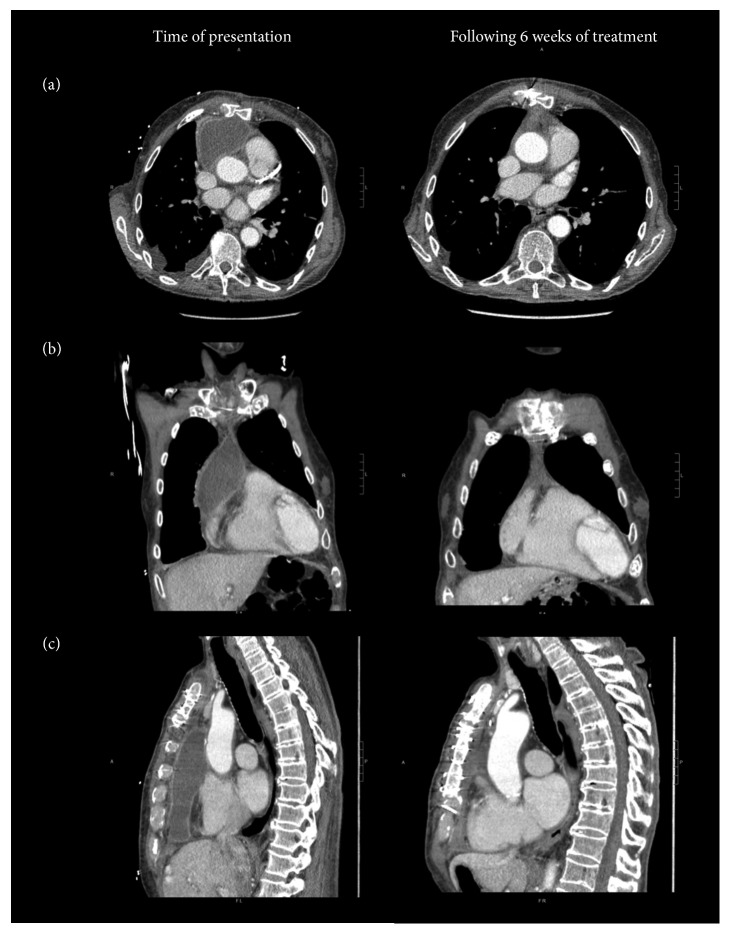
Computed tomography imaging with intravenous contrast showing nontyphoidal *Salmonella* mediastinal abscess. Panel (a) shows axial views, panel (b) shows coronal views, and panel (c) shows sagittal views at the time of presentation (left) and following 6 weeks of therapy with intravenous ceftriaxone (right).

Computed tomography of the chest demonstrated a 13.6-centimeter rim-enhancing fluid collection in the anterior mediastinum with multiple small foci of gas, an additional 2.5-centimeter collection between the clavicular heads, and new skin thickening and soft-tissue stranding in the anterior chest wall concerning for soft-tissue infection (Figure 2). Blood cultures were drawn, and treatment was initiated with intravenous vancomycin and piperacillin-tazobactam. He was admitted to the cardiothoracic surgery service, and on hospital day two, underwent washout of the collection, during which time his midline incision was reopened, and two pockets of pus were identified and evacuated. A wound vacutainer system was placed. On hospital day three, one of the two sets of admission blood cultures and two intraoperative wound cultures grew Gram-negative rods. Vancomycin was discontinued, and the patient was maintained on piperacillin-tazobactam. On hospital day 4, the organism was identified as *Salmonella* group D. Susceptibility testing showed that the isolate was susceptible to ampicillin (MIC < 8 *μ*g/mL), ceftriaxone (MIC < 0.5 *μ*g/mL), and trimethoprim-sulfamethoxazole (MIC < 2 *μ*g/mL) but had intermediate susceptibility to ciprofloxacin (MIC 0.12 *μ*g/mL). The infectious disease service was consulted and recommended switching piperacillin-tazobactam to ceftriaxone.

The patient had no personal or family history of unusual infections. He was a 30 pack-year former smoker (quit in 1980), drank alcohol in moderation, and had no history of illicit substance use. He had traveled to Switzerland several years prior and had remote travel to Southeast Asia. He reported consumption of raw fish, raw beef, and incompletely cooked eggs. The patient had a dog, but no exposure to chickens or other birds. Notably, the patient's son had recently returned from college to help his father during the postoperative period and had brought with him a pet snake. Although the patient did not specifically interact with the snake, he reported that his son had prepared food for him after handling the animal.

The patient required two additional debridements, and during the final procedure, the sternum was reopened to confirm no further infection of the mediastinum by direct visualization. Transthoracic and transesophageal echocardiography were performed and showed no evidence of valvular infection or involvement of the aorta. His wound was closed, and he was discharged in good clinical condition to continue his antibiotic therapy as an outpatient, with a planned course of intravenous ceftriaxone 2 grams daily for a total of six weeks. A repeat chest CT after completion of therapy showed no evidence of aortic involvement, a small amount of residual fluid, and sternal bony changes concerning for remodeling versus infection (Figure 2). Because of the remaining fluid and concern for possible sternal osteomyelitis, he was treated with oral amoxicillin consolidation therapy for an additional 4 weeks, although ultimately the CT findings were thought to be more likely related to poststernotomy changes. Due to intolerance, amoxicillin was changed to cefixime; his course was complicated by an episode of mild *Clostridium difficile* infection. A C-reactive protein downtrended from 190 mg/L to 5.5 mg/L. At the time of writing, the patient has been off antibiotic therapy for four months and continues to do well.

## 2. Discussion

Nontyphoidal *Salmonella* (NTS) are Gram-negative bacilli that cause a range of infectious syndromes in humans. Infection occurs via person-to-person transmission, food-borne infection, or animal exposure. Most often, NTS causes a self-limited gastroenteritis characterized by intestinal inflammation and diarrhea. In some proportion of affected individuals, bacteria gain access to the circulatory system with resulting bacteremia. NTS bacteremia is more prevalent in sub-Saharan Africa and is more common in individuals at the extremes of age or who are immunosuppressed. Following gastrointestinal infection, it is relatively common for patients to exhibit short-term NTS shedding in the stool and a small subset of individuals may go on to be chronic, asymptomatic NTS carriers [1]. Controversy exists regarding the relationship between antibiotic treatment and duration of carriage [1].

NTS may also present as a focal infection. In a large study of NTS infections in New York, investigators noted that 7.4% of cases presented with focal infections rather than diarrhea [2]. In that study, most focal infections were those related to the gastrointestinal tract, e.g., appendicitis or cholecystitis. A study of all focal NTS infections in a Spanish hospital over ten years demonstrated a similar predilection for the gastrointestinal and genitourinary systems, which together accounted for 20 out of 35 cases [3].

Mediastinal infections with NTS are rare and generally involve a combination of preexisting tissue disruption, unusually susceptible hosts, animal exposure, and/or documented positive stool cultures. To our knowledge, only 12 cases of *Salmonella* mediastinal infection have been reported, including this current case (Table 1). Only three cases occurred in the absence of preexisting tissue disruption: one patient was on immunosuppressive agents and had exposure to chickens [4], the second had hairy cell leukemia [5], and the third was immunocompetent but had recently sustained a facial bite from a raccoon [6]. Four cases were reported demonstrating NTS infection of abnormal thoracic tissues, including NTS abscess development within known [7–9] or suspected [10] thymomas.

Postoperative mediastinal NTS infection is even more rarely reported, and we identified only four prior cases in addition to our patient, three of which occurred in young children (Table 1). In the only previously reported adult case, a patient who had undergone valve replacement surgery developed NTS bacteremia, aortic dissection, and mediastinitis in the postoperative period; this patient had a stool culture that was positive for NTS [11]. Similarly, a child developed NTS mediastinitis after undergoing correction of transposition of the great arteries, and this patient had both documented diarrhea and a positive stool culture [12]. Two cases of postoperative NTS mediastinal infection in children were reported by a French hospital, and both patients had negative stool cultures and no recorded diarrheal illness. One child had developed mediastinitis following pericardiocentesis, while the other had undergone cardiac surgery for the correction of an atrioventricular connection [13]. Of note, of the nine cases reported in the literature in which stool was tested for NTS (Table 1), only three patients had positive cultures. It is possible that this number may underestimate patients who had gastrointestinal carriage prior to developing mediastinitis, first because shedding of NTS in stool is generally a transient phenomenon [1], and second because antibiotic use may have been initiated prior to stool sampling.

The patient reported in this case study had many of the predisposing factors for NTS infection described above (detailed in Table 1). He had mediastinal tissue disruption (his recent surgery) and exposure to both undercooked food and to a snake, both possible sources of infection. Snakes and other squamates are frequently colonized with NTS, and contact with these animals in the domestic setting has been linked with an increasing number of human infections [14]. Our patient had negative stool cultures, but it should be noted that stool samples were taken after initiation of antibiotics. Finally, although our patient was not on immunosuppressive medications, both his advanced age and his recent cardiac surgery may have increased his susceptibility to this pathogen. Experiments in a mouse model of NTS have demonstrated increased tissue colonization and weight loss in elderly mice; this increased susceptibility may be mediated by defects in the cytokine response to the pathogen [15]. Surgery itself has been linked to immunosuppression through multiple mechanisms; this patient's recent cardiac surgery likely also contributed to the development of infection [16].

We hypothesize that the patient developed an asymptomatic gastrointestinal infection with NTS after exposure to undercooked food or his son's pet snake, became transiently bacteremic, and then seeded the tissues that were vulnerable following the surgical procedure. As this collection expanded, he likely developed recurrent bacteremia, which was detected on his admission cultures. An alternative explanation would invoke direct infection of the patient's mediastinum during the surgical procedure, but infection secondary to intraoperative contamination would be much more likely to involve usual components of skin flora (*Staphylococcus* or *Streptococcus* species) rather than the comparatively rare, gastrointestinal pathogen *Salmonella.*

NTS has a predilection for the endovascular environment and is one of the two most common pathogens (with *S. aureus)* isolated from mycotic aortic aneurysms [17]. Given his recent valve replacement and NTS bacteremia, a major clinical concern in this case was the risk that the patient might develop this complication. Our patient's age and sex may possibly have put him at additional risk. A study in a Jerusalem hospital noted that in 105 patients with documented NTS bacteremia, three developed aortitis, and all three were male and over the age of 65 [18]. Fortunately, however, a TEE showed no evidence of aortitis, and imaging did not show evidence of vascular involvement; our patient's overall improving clinical status was inconsistent with active aortic infection.

Fluoroquinolones have previously been the drug of choice for NTS infection, but antibiotic resistance is an increasing problem, as was seen in this case. According to the CDC National Antimicrobial Resistance Monitoring System for Enteric Bacteria (NARMS), resistance to fluoroquinolones in NTS isolates in the USA exceeded 4% as of 2014 [19]. Our patient's geographic location may have increased his risk. NARMS reports that, in California in 2015, fluoroquinolone resistance was noted in 9/63 isolates (14.3%) [20]. Ceftriaxone resistance, however, has not been reported in California [20]. Given these data, our patient's antimicrobial susceptibility results, and the severity of his illness, we decided to treat with a full 6-week course of ceftriaxone, followed by 4 weeks of oral beta-lactam consolidation therapy.

## 3. Conclusion

We have described an unusual case of a postoperative NTS mediastinal abscess following aortic valve replacement in an elderly man with potential food and animal exposure to nontyphoidal *Salmonella*. Key educational points include the epidemiologic characteristics of NTS, the risk factors for development of postoperative NTS infections, and the complications for which such patients should be monitored. This case also highlights the need to thoroughly cook eggs, meat, and fish and to maintain hand hygiene when handling pets, especially in elderly or immunocompromised people.

## Figures and Tables

**Table 1:** Comparison of the current case to previously reported cases of nontyphoidal *Salmonella* mediastinitis.

Ref	Author	Year	Patient age	Patient sex	Mediastinal tissue disruption	Animal exposure	Preexisting immunosuppression	Preceding diarrheal illness	Stool culture	Treatment	Outcome
*No previous tissue disruption*											
4	Joshua et al.	2003	34 y	F	None	Domestic chickens	Immunosuppressant drugs	Yes	Positive	Drainage; 14 d IV ciprofloxacin followed by 14 d PO ciprofloxacin	Recovery
5	Tilly et al.	1985	53 y	M	None	Not reported	Hairy cell leukemia	No	Negative	Drainage; 7 d trimethoprim-sulfamethoxazole followed by ampicillin (dns)	Recovery
6	Marsh et al.	1984	14 y	M	None	Wild raccoon bite	None	No	Not stated	Drainage; IV cefamandole (dns) followed by 21 d cephalexin	Recovery
*Preexisting abnormal thoracic tissue*											
7	Carter et al.	2005	53 y	F	Thymoma	Not reported	None	No	Negative	Resection; ceftriaxone (dns)	Recovery
8	Lo et al.	2015	59 y	M	Thymoma	Not reported	None	No	Not stated	Resection; cefipime (dns)	Recovery
9	Saheer et al.	2015	21 y	M	Thymoma	Not reported	None	No	Negative	Drainage, ceftriaxone (dns)	Recovery
10	Snider et al.	1993	66 y	F	Mass (possible thymoma)	Not reported	None	Yes	Negative	Drainage; ampicillin (dns)	Recovery
*Preceding surgical intervention*											
11	Fernandez-Ayala et al.	2003	70 y	F	Surgery	Not reported	None	No	Positive	Drainage; 6 w ampicillin and ciprofloxacin	Sudden death 30d postdischarge
12	Aherne	1984	20 m	M	Surgery	Not reported	None	Yes	Positive	Drainage; 21 d chloramphenicol	Recovery
13	Nordmann et al.	1994	5 m	M	Surgery	Not reported	None	No	Negative	Drainage; 21 d ceftriaxone and pefloxacin, then 1 m ceftriaxone	Recovery
13	Nordmann et al.	1994	1 y	M	Pericardiocentesis	Not reported	None	No	Negative	Drainage; 1 m pefloxacin and imipenem/cilastatin, then 1 m pefloxacin	Recovery
Current case			86 y	M	Surgery	Domestic snake	None	No	Negative	Drainage; 6 w ceftriaxone, then 4w beta-lactam	Recovery

Abbreviations: y = years, m = months, w = weeks, d = days, dns = duration not specified.
